# SOD1 is a novel prognostic biomarker of acute kidney injury following cardiothoracic surgery

**DOI:** 10.1186/s12882-023-03350-8

**Published:** 2023-10-11

**Authors:** Joseph H. Holthoff, Yanping Harville, Christian Herzog, Luis A. Juncos, Nithin Karakala, John M. Arthur

**Affiliations:** 1 Department of Nephrology, University of Arkansas for Medical Sciences, 4301 W. Markham St. #501, Little Rock, AR 72205 USA; 2 Division of Nephrology, Central Arkansas Veterans Healthcare System, Little Rock, AR 72205 USA

**Keywords:** Acute kidney injury, Biomarkers, SOD1, Renal replacement therapy

## Abstract

**Background:**

Acute kidney injury (AKI) is a major burden among hospitalized and critical care patients. Among hospitalized patients that progress to severe AKI there is increased risk for morbidity, mortality, and the need for renal replacement therapy (RRT). As there are no specific treatments for AKI, the discovery of novel biomarkers that predict the progression of AKI may aid in timely implementation of supportive care to improve outcomes.

**Methods:**

We collected urine from 204 patients that developed Stage 1 AKI by AKIN criteria within 72 h following cardiothoracic surgery. Urine samples were collected at the time of the initial diagnosis of AKI and stored at ^−^80° C. Among the 204 patients, 25 progressed to a composite primary outcome of Stage 3 AKI, requirement of RRT, or 30-day mortality. The remaining 179 patients did not progress beyond Stage 2 AKI and were considered controls. Urinary concentrations of SOD1 and SOD1 activity were measured following collection of all samples. Samples were thawed and urinary superoxide dismutase 1 (SOD1) concentrations were measured by sandwich ELISA and urinary SOD1 activity was measured through a commercially available colorimetric assay.

**Results:**

Urinary concentrations of SOD1 were significantly elevated (67.0 ± 10.1 VS 880.3 ± 228.8 ng/ml, *p* < 0.0001) in patients that progressed to severe AKI and were able to predict the progression to severe AKI (AUC – 0.85, *p* < 0.0001). Furthermore, total SOD activity also increased in the urine of patients that required RRT (77.6% VS 49.81% median inhibition, *p* < *0.01*) and was able to predict the need for RRT (AUC: 0.83, *p* < 0.01).

**Conclusion:**

These findings show that urinary SOD1 concentrations and SOD activity are novel prognostic biomarkers for severe AKI following cardiothoracic surgery.

## Background

AKI is defined as a sudden rise in serum creatinine or a decline in urine output. The incidence of AKI is increasing [1, 2] and is responsible for a higher inpatient mortality rate and increased economic burden on the health care systems [3–5]. AKI affects up to 20% of hospitalized patients and the mortality rate for patients that progress to severe AKI requiring renal replacement therapy (RRT) approaches 50% [2–4]. Despite this huge impact on the healthcare system, there remains no specific and targeted interventions for AKI despite numerous clinical trials and decades of preclinical studies [3, 6].

The current definition of AKI has severe limitations [7, 8]. The use of serum creatinine and urine output as diagnostic criteria for AKI provide little information regarding disease progression. The ability to identify novel biomarker that predict the progression to severe AKI could lead to more timely interventions and improve clinical outcomes, enrich clinical trial enrollment, and uncover novel mechanistic targets that may ultimately lead to more specific treatments for AKI [4, 9, 10].

Over the last decade, several molecules have been proposed as potential biomarkers of AKI [4, 11–13]. A planned prospective observational analysis of the Early Intervention with Erythropoietin trial (EARLYARF) evaluated urinary γ-glutamyl transpeptidase (GGT), alkaline phosphatase, cystatin C (Cys-C), kidney injury molecule 1 (KIM-1), and neutrophil-gelatinase-associated lipocalin (NGAL) for the ability to diagnose AKI early in the disease process and predict the progression to severe AKI in a cohort of 529 intensive care patients [14, 15]. All molecules showed limited efficacy for the early diagnosis of AKI at the time of admission to the intensive care unit (ICU); while NGAL, Cys-C, and IL-18 were able to predict the need for renal replacement therapy. Upon stratification for time, the predictive value of each molecule improved suggesting there are dynamic changes in biomarker expression as it relates temporally to the timing of the initial insulting injury. The Sapphire study validated two molecules implicated in G1 cell cycle arrest as biomarkers for the progression to severe AKI in a heterogenous cohort of 728 ICU patients: Tissue inhibitor of metalloproteinases-2 (TIMP-2) and insulin-like growth factor binding protein 7 (IGFBP7) [11]. The product of [TIMP-2]*[IGFBP7] has been validated to predict the progression to severe AKI [11, 16, 17]. However, to date all potential biomarkers of AKI have failed to display the diagnostic sensitivity and specificity which is required to be reliably implemented in wide spread clinical practice.

There are several hurdles that impede the discovery of novel prognostic biomarkers for AKI including a heterogenous patient population, multifactorial causative insults, patient comorbidities, and a poor understanding of the pathogenesis of AKI. These same obstacles also hinder the translation of novel therapeutic approaches from animal studies to clinical therapy [9]. The discovery of functional biomarkers of AKI not only holds promise for early clinical diagnosis and prognostication, but could also provide physiological insight into the complex molecular mechanisms of AKI [11, 14, 17, 18]. A more in depth understanding of the molecular mechanisms of AKI could overcome the hinderances of translation and lead to new therapeutic targets for AKI.

We have previously identified 30 candidate prognostic biomarkers of AKI in a discovery cohort of patients that developed AKI following cardiothoracic surgery using liquid chromatography/tandem mass spectrometry LC–MS/MS. SOD1 was one of the more promising biomarkers from the discovery cohort [18]. Thus, we decided to proceed with the validation of SOD1 as a novel prognostic biomarker of AKI in a larger patient cohort presented here. SOD1 is an endogenous antioxidant enzyme localized to the cytosol of cells. It serves to catalyze the conversion of superoxide anion to molecular oxygen (O_2_) and hydrogen peroxide (H_2_O_2_). In the kidney, SOD1 is found mainly in the mitochondrial laden renal tubular epithelial cells [19]. In this study we show that both urinary SOD1 concentration and total SOD activity are dramatically increased in patients that progress to severe AKI following cardiothoracic surgery. These studies not only implicate urinary SOD1 concentration and activity as novel prognostic biomarkers of AKI, but could lay the foundation for future studies to unravel the mechanistic role that SOD1 plays in AKI, ultimately leading to novel therapeutic approaches.

## Methods

### Collection of urine samples

Urine samples were obtained consecutively from 322 patients who underwent cardiothoracic surgery at SAKInet institutions between August 1^st^, 2008 and June 1^st^, 2012 within 48 hours post-operatively [18, 20]. Prospective written informed consent was obtained in accordance with the institutional review board at the University of Arkansas for Medical Sciences (#205605). Urine samples were collected followed by centrifugation, the addition of protease inhibitors, and then stored at -80°C. Inclusion criteria was defined as the development of AKIN stage 1 AKI within 72 hours post-operatively following surgery of the heart or ascending aorta. Exclusion criteria for enrollment were patients with end stage kidney disease (ESKD), chronic kidney disease (defined as a baseline serum creatinine > 3.0 mg/dl), or AKIN stage 2 AKI or higher at the time of collection. Of the 322 samples collected from enrolled patients, 108 samples were excluded due to the following: insufficient sample (*n* = 21), already KDIGO stage 2 AKI or higher at time of collection (*n* = 25), or the patient did not ultimately develop AKI within 72 hours following surgery (*n* = 62). These exclusion left a total of 204 remaining patients available for the validation study (181 controls and 25 that eventually progressed to severe AKI). Patients were followed until hospital discharge or death and AKI was staged according to the maximum increase in serum creatinine using the AKIN classification.

### Definition of the primary outcome

The primary outcome was initially defined at the start of the study as a composite of the progression to stage 3 AKI, the need for RRT, and/or 30-day mortality as a marker of severe clinical illness, including severe renal injury. It should be noted all patient that experienced death within 30 days also met inclusion criteria by the development of stage 3 AKI or the need for RRT. There were 25 patients that progressed to meet our primary outcome. The initiation of RRT and the management of AKI prior to the initiation of RRT was at the discretion of the treating physician. There were 179 patients that did not progress beyond stage 2 AKI and these patients were considered controls.

### Measurement of urine SOD1 and NGAL concentrations

Urine samples were thawed in a 37°C water bath and commercially available sandwich Elisa was used to quantify urine SOD1 concentrations (eBiosciences, Waltham, MA) and urine NGAL concentrations (R&D Systems, Minneapolis, MN) according to the manufacturer’s protocol. Each sample was performed in duplicates.

### Measurement of urine SOD activity

Fifteen patients in our cohort required RRT. To determine the ability of SOD activity to predict the need for RRT, patients that required RRT were matched with control patient with the same serum creatinine at the time of collection. SOD activity of urine samples were measured using a commercially available kit (Sigma-Aldrich, St. Louis MO) according to the manufacturer’s directions. Briefly, urine samples were thawed in a 37°C water bath and then centrifuged at 700 g for 10 minutes. Supernatant was diluted 1:10 with reaction buffer provided by the manufacturer (Sigma-Aldrich, St. Louis Mo.). Tetrazolium salt [2-(4-iodopehyl)-3-(4-nitrophenyl)-5-(2,4-disulfophenyl)-2-tetrazolium, monosodium salt] supplied by the manufacturer produces a formazan dye upon reduction by superoxide anions to generate a colorimetric change that can be measure at 440 nm. The SOD1 activity can be quantified as the percent inhibition of the colorimetric change. Samples were performed in duplicates.

### Statistical analysis

Data was analyzed and figures generated using Graphpad Prism (9.2.0). Unpaired t-test with were used to analyze data when two groups were present and one-way ANOVA was used for comparison of multiple groups. A Grubb’s test was used to identify data points which were significant outliers. Statistical significance was considered (*p* < 0.05). Receiver operator characteristics curves (ROC) were generated and analyzed using Graphpad Prism (9.2.0).

## Results

### Subject enrollment

**Fig. 1:**
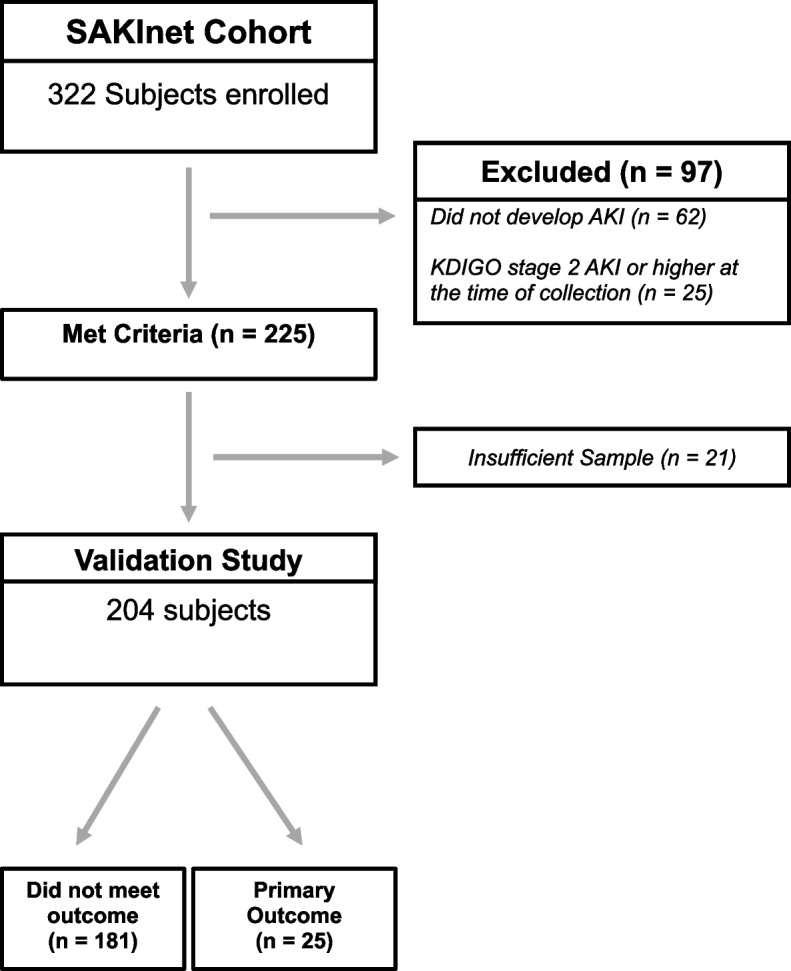
Study design and patient enrollment: A total of 322 patients were enrolled in the study. Patients that did not develop AKI (*n* = 62), had already progressed to KDIGO stage 2 AKI (*n* = 25) at the time of sample collection, or had insufficient sample collection (*n* = 21) were excluded. The remaining 204 patients were analyzed in the validation study

We enrolled a total of 322 patients in our cohort (Fig. 1). Patients were excluded if they did not develop AKI, were already at least AKI stage 2 at the time of collection, or if there was not sufficient sample to be processed. We collected urine samples from 204 patients that developed stage 1 AKI within the first 72 h following cardiothoracic surgery. Patients were followed throughout the duration of hospitalization and classified as “controls” (did not progress beyond AKIN stage 2 AKI) or “progressors” (developed the composite outcome of AKIN stage 3 AKI, need for renal replacement therapy, or death within 30 days). The initiation of renal replacement therapy was at the discretion of the treating physician.

### Patient characteristics

Data describing patient characteristics can be found in Table 1. There was no difference between the control and progressor patient cohorts in regards to age (66.1 ± 0.9 VS 69.6 ± 2.9, *p* = *0.197*), weight (89.5 ± 1.5 VS 91.7 ± 5.7 kg, *p* = *0.628*), gender (66.5 VS 64% males, *p* = *0.665*), or ethnicity (68.7 VS 76% Caucasian, *p* > *0.461*). There was no difference between controls and progressors regarding pre-existing medical comorbidities including diabetes (39 VS 44%, *p* > *0.603*), systolic heart failure with ejection fraction < 35% (16.2% VS 32%, *p* = *0.055)*, or baseline serum creatinine (1.17 ± 0.03 VS 1.32 ± 0.08 mg/dl, *p* = *0.08*). Surgical parameters including history of previous cardiac surgery (40 VS 44%, *p* = 0.633), the need for pre-operative IABP (10.1% VS 16%, *p* = 0.379), the mean cardiac bypass time (134.5 ± 6.2 VS 139.9 ± 20.5 min, *p* = 0.7655), or type of surgical procedure (Table 2) was not statistically different between controls and patients that progressed to severe AKI. There was no difference in the average time of sample collection between the two groups (26.3 ± 1.0 VS 27.5 ± 2.8 h, *p* = 0.694).

**Table 1:** Patient demographics

	**Controls (189)**	**Progressors (25)**	***P – Value***
**Age (years)**	66.1 (± 0.92)	69.6 (± 2.91)	*P* = *0.197*
**Weight (kg)**	89.48 (± 1.56)	91.74 (± 5.72)	*P* = *0.628*
**Gender (% male)**	124 (66.5%)	13 (64%)	*P* = *0.665*
**Ethnicity (% White)**	123 (68.7%)	19 (76%)	*P* = *0.461*
**Diabetes**	69 (39%)	8 (44%)	*P* = *0.603*
**Baseline creatinine (mg/dl)**	1.17 (± 0.03)	1.32 (± 0.08)	*P* = *0.079*
**Ejection Fraction < 35% (%)**	29 (16.2%)	8 (32%)	*P* = *0.055*
**Previous cardiac surgery (%)**	70 (40%)	8 (32%)	*P* = *0.633*
**Pre-operative IABP (%)**	18 (10.1%)	4 (16%)	*P* = *0.379*
**Bypass time (mins)**	134.5 (± 6.2)	139.9 (± 20.5)	*P* = *0.7655*
**Mean collection time after surgery (hours)**	26.34 (± 0.97)	27.45 (± 2.77)	*P* = *0.694*

Data are expressed as mean ± SEM for parametrically distributed data. Non-parametric data is expressed as % of entire patient cohort. Data was analyzed using unpaired T-test. A *p-value* < 0.05 was considered statistically significant

**Table 2:** Type of cardiothoracic surgery

	**Controls (189)**	**Progressors (25)**	***P – Value***
**Coronary artery bypass graft (CABG)**	121 (67.6%)	15 (60%)	*P* = 0.499
**Mitral valve repair**	44 (24.6%)	3 (12%)	*P* = 0.209
**Aortic valve repair**	39 (21.8%)	7 (28%)	*P* = 0.609
**Tricuspid valve repair**	8 (4.47%)	1 (4%)	*P* = 0.919
**MAZE procedure**	2 (1.1%)	1 (4%)	*P* = 0.326
**Thoracic aneurysm repair**	3 (1.7%)	1 (4%)	*P* = 0.410

Data are expressed as mean ± SEM for parametrically distributed data. Non-parametric data is expressed as % of entire patient cohort. Data was analyzed using unpaired T-test. A *p-value* < 0.05 was considered statistically significant

### Urine SOD1 concentrations

**Fig. 2:**
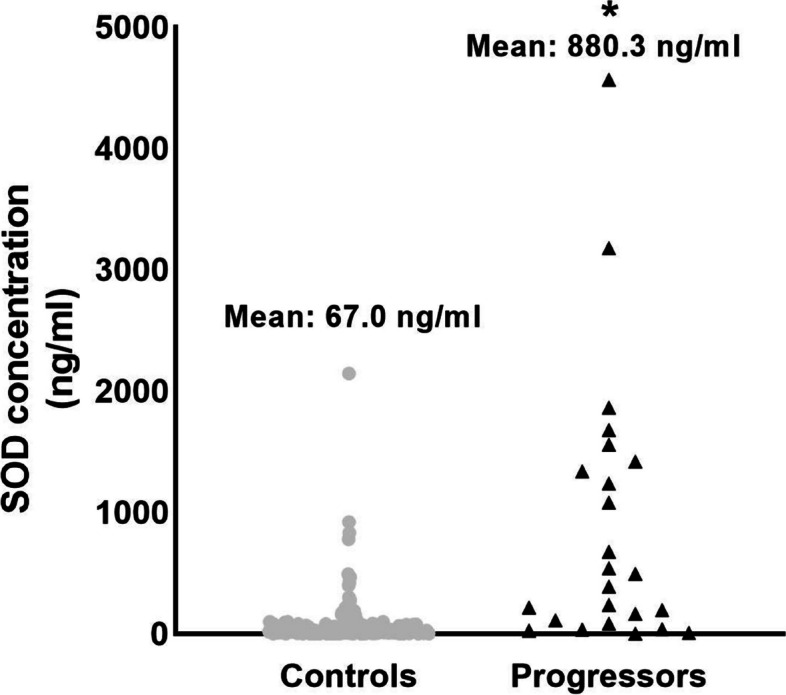
Urine SOD1 concentrations. Urinary concentrations of SOD1 were significantly elevated in patients that progressed to the primary endpoint (67.0 ± 10.1 VS 880.3 ± 228.8 ng/ml, * indicates statistical difference from control by unpaired T-test, *p* < 0.0001)

**Fig. 3:**
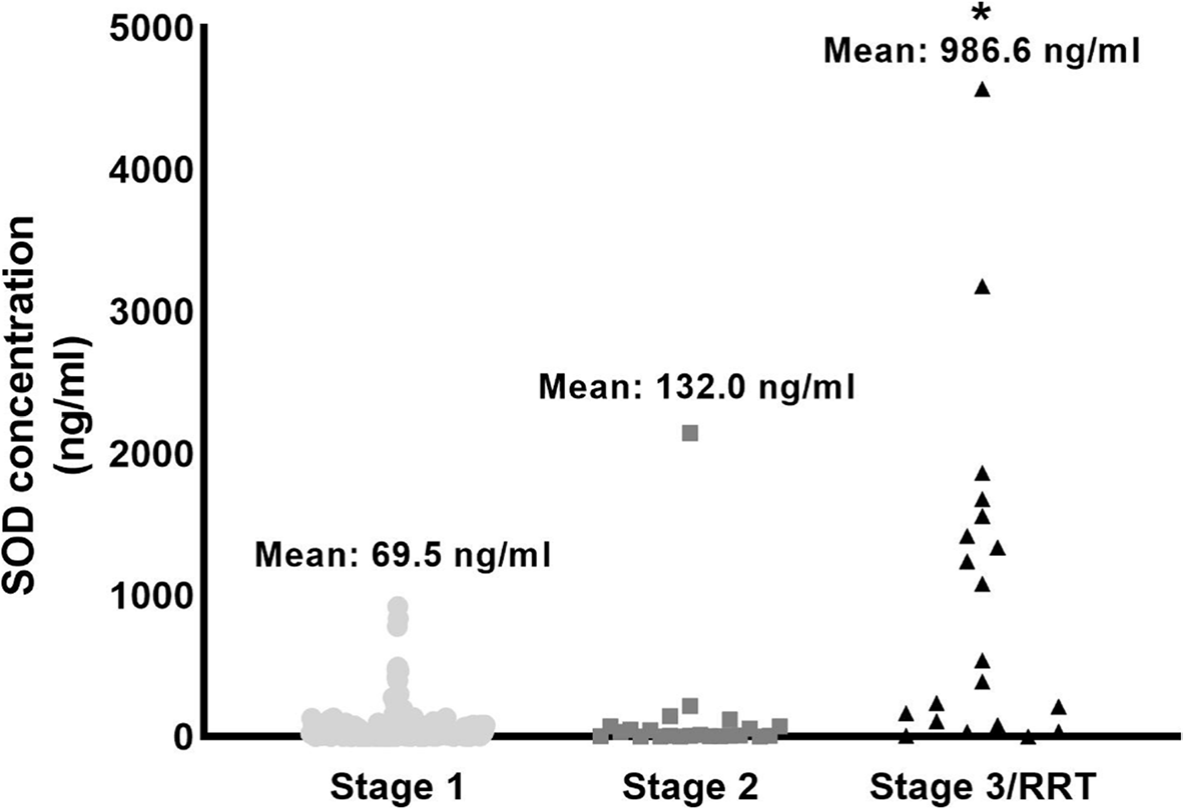
Urinary SOD1 concentration by KDIGO AKI staging. Urinary concentrations of SOD1 were separated by stage of maximum AKI progression. The was a progressive increase in the mean urinary SOD1 concentrations with each stage of AKI: stage 1 (69.5 ng/ml ± 11.2 ng/ml, *n* = 158) VS stage 2: (132 ng/ml ± 92.1 ng/ml, *n* = 23) VS stage 3 AKI (986.8 ± 267.9 ng/ml, *n* = 20), * indicates significant difference from stage 1 AKI *p* < 0.0001 by one-way ANOVA

To validate our previously reported proteomic data [18], urinary concentrations of SOD1 were measured by sandwich Elisa (Fig. 2). Patients that progressed to severe AKI showed significantly higher concentrations of urinary SOD1 compared to controls (880.3 ± 228.8 ng/ml VS 67.0 ± 10.1 ng/ml, *p* < *0.0001*). Furthermore, urinary SOD concentrations increased progressively with each stage of AKI (stage 1: 69.5 ng/ml ± 11.2 ng/ml, *n* = 158 VS stage 2: 132 ng/ml ± 92.1 ng/ml, *n* = 23 VS stage 3 AKI 986.8 ± 267.9 ng/ml, *n* = 20, *p* < 0.001 for Stage 3 compared to Stage 1 AKI) (Fig. 3).

### Urine SOD1 concentration ROC

**Fig. 4:**
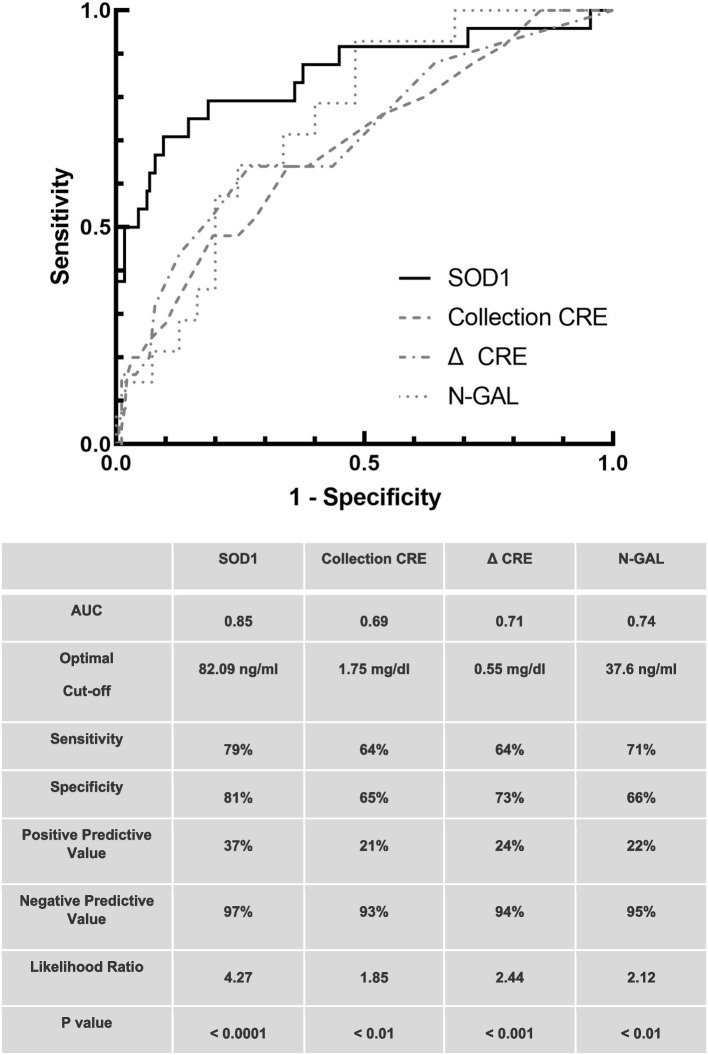
Comparison of prognostic value for urinary SOD1 and serum creatinine. Urinary concentrations of SOD1 (AUC: 0.85) showed improved ability to predict the progression to severe AKI when compared with serum creatinine concentrations at the time of sample collection (AUC: 0.69), the change in serum creatine from collection to peak creatinine value (AUC: 0.71), and the established AKI biomarker NGAL (AUC: 0.74). Optimal cutoff values, sensitivity, specificity, positive predictive values, negative predictive values, likelihood ratio, and *p*-values are presented for each biomarker

As we found that urine concentrations of SOD1 were significantly increased in patients that progressed to severe AKI, we generated ROC curves to determine the prognostic ability of urinary SOD1 concentrations to predict the progression to severe AKI. The area under the curve (AUC) was 0.85 (95% CI: 0.75 – 0.95, *p* < 0.0001) (Fig. 4). Using a cutoff value for urinary SOD concentration of 82.09 ng/ml, we were able to predict the progression to severe AKI with high sensitivity and specificity (sensitivity: 79%, specificity: 81%, likelihood ratio: 4.27). The positive predictive value of urinary SOD1 concentration was (37%) and the negative predictive value was (97%). Although serum creatinine at the time of sample collection (AUC: 0.69, *p* < 0.01), the change to maximum serum creatinine (AUC: 0.71, *p* < 0.001), and urinary NGAL concentrations (AUC: 0.74, *p* < 0.01) were also able to statistically predict the progression to severe AKI, urinary SOD1 concentrations showed better performance for all diagnostic parameters (Fig. 4).

### Urine SOD1 activity

**Fig. 5:**
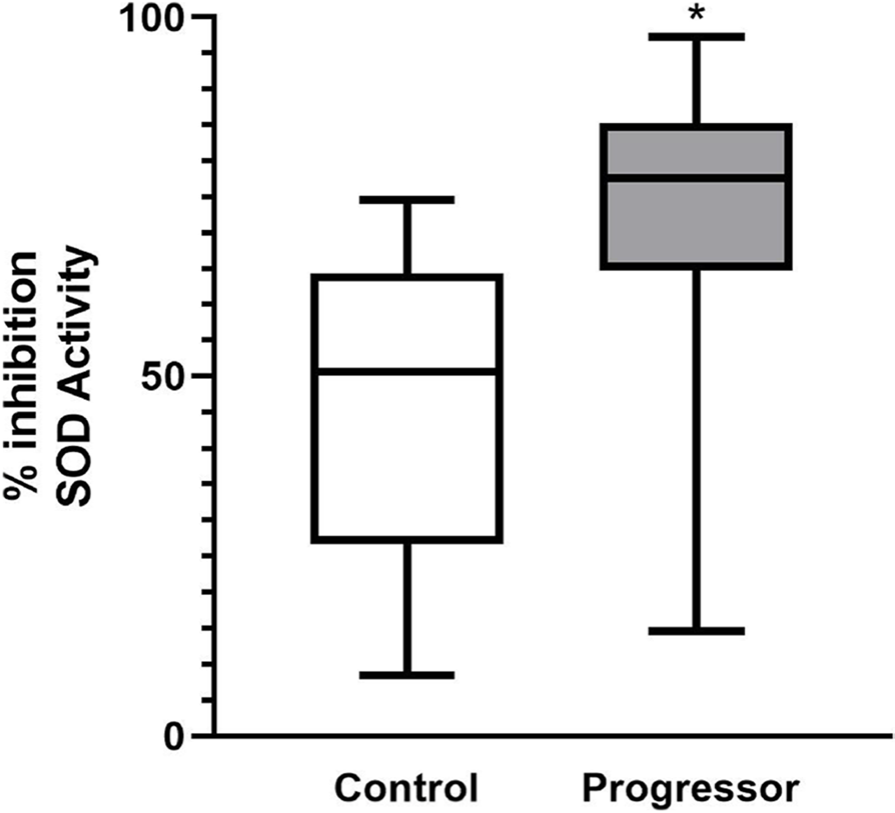
Urine SOD activity: Urinary SOD activity is significantly higher in patient that progress to more severe AKI (77.6% VS 49.81% median inhibition, *p* < *0.01*). Enclosed boxes represent the interquartile range of the data with bars and whiskers representing the range of minimum to maximum values. The horizontal line within the enclosed boxes represents the median value. * represents statistical difference from control group by unpaired T-test

**Fig. 6:**
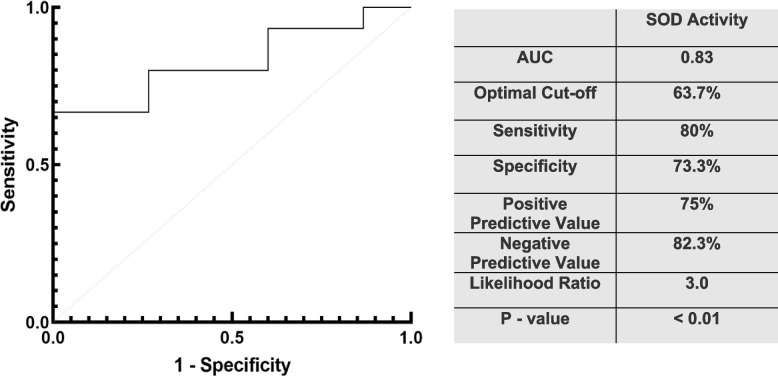
ROC curve of urine SOD activity: Urinary SOD activity was able to predict the need for RRT (*n* = 15) compared to patient that didn’t progress beyond stage 1 AKI (*n* = 15, AUC: 0.83, *p* < 0.01). The optimal cutoff value, sensitivity, specificity, positive predictive value, negative predictive value, likelihood ratio, and *p* value are also presented

The urinary SOD activity was measured to determine if urinary SOD1 retained physiological activity, and could possibly implicate a mechanistic role for urinary SOD1 in AKI. Patients that progressed to require RRT (*n* = 15) were matched to controls with the same serum creatinine at the time of collection who did not progress beyond stage 1 AKI (*n* = 15). Urine of patients that progressed to require RRT displayed higher levels of SOD activity (77.6% VS 50.6% median inhibition, *p* < *0.001*) as expressed by the percent inhibition of tetrazolium salt reduced by superoxide anion (Fig. 5). Using an optimal cut-off value of 63.7% inhibition, urinary SOD activity was able to predict the need for RRT (AUC: 0.83, 95% CI: 67.5% – 98.4%, *p* < 0.01) with high sensitivity (80%), specificity (73.3%), and likelihood ratio [3]. Positive predictive value and negative predictive value were 75% and 82.3%, respectively (Fig. 6).

## Discussion

In this study, we identified urinary concentration of SOD1 and urinary activity of SOD as novel prognostic biomarkers for the progression to severe AKI. Our validation of urinary SOD1 concentrations and SOD activity to predict the progression to severe AKI was very good with AUC values of 0.85 and 0.83, respectively. Urinary concentrations of SOD1 and SOD activity showed improved ability to predict the progression to severe AKI compared to creatinine at the time of collection and the total change in creatinine to peak. These value are improved when compared against those reported by others for traditional biomarkers of AKI including NGAL, KIM-1, and IL-18 [11, 21] and comparable to those reported for [TIMP-2 * IGFBP-7] [11, 16, 17]. The Sapphire study validated [TIMP-2 * IGFBP-7] in a heterogenous cohort of 728 patients critically ill patients [11] that progressed to KDIGO stage 2–3 AKI. The AUC for [TIMP-2 * IGFBP-7] was 0.80 and was superior to more traditional biomarkers. The ability to identify patients at risk of progression to severe AKI from a myriad of insulting etiologies is highly clinically relevant, as the etiology of AKI in clinical practice is often not known or may be multifactorial [1, 22, 23]. Our study enrolled a smaller patient cohort (*n* = 204) and was limited to only patients that developed AKI following cardiothoracic surgery. However, cardiothoracic surgery remains a major risk for the development of AKI and is associated with a high incidence of perioperative AKI [24–26]. Our findings hold much promise for early identification of patients at risk to develop severe AKI following cardiothoracic surgery and may allow for implementation of earlier supportive care.

Superoxide dismutase(s) are a group of metalloproteins that function as endogenous antioxidants by neutralizing superoxide radicals to form molecular oxygen and hydrogen peroxide. Copper-Zinc superoxide dismutase (SOD1) is one of 3 isoforms of SOD and is localized to the cytosol of cells, while SOD2 is localized to the mitochondria, and SOD3 is found predominantly in the extracellular space [27]. In the kidney, SOD1 is found mainly in the renal tubular epithelial cells and accounts for 80% of total renal SOD activity [19]. SOD1 has been implicated in animal models to play a mechanistic role in various human health conditions [28] including amyotrophic lateral sclerosis (ALS) [29–31], cardiac ischemia [32], and even AKI [33]. Ischemia/reperfusion induced AKI in mice is associated with depleted SOD1 concentrations in kidney tissue homogenates [34] and SOD1 knockout mice suffer more profound AKI compared to their wild type counterparts [35]. However, the contribution of SOD1 during AKI in humans is not known.

Our finding that urinary SOD1 concentrations and total urinary SOD activity are both increased in patients that develop severe AKI following cardiothoracic surgery is novel. Furthermore, these biomarkers were able to accurately predict patients likely to progress to severe AKI. Others have shown that SOD concentrations and antioxidant activity decrease in the serum of patients undergoing chronic renal replacement therapy [36, 37] and patients with chronic kidney disease [36, 38]. However, these studies provide no insight into the role of SOD1 during AKI. To our knowledge we are the first to report that urinary SOD1 concentrations and total urine SOD activity are prognostic biomarkers of AKI. *Costa *et al. reported that erythrocyte SOD1 activity was decreased in critically ill patients that developed septic AKI [39]. However, these findings are likely not specific to SOD1 as extracellular SOD3 is the predominant isoform present in human serum and SOD activity assays are unable to differentiate contributions between multiple isoforms [27]. Although we cannot rule out the possibility that the increased urinary SOD activity in our patient cohort has contributions from other isoforms of SOD, it is known that SOD1 makes up 80% of total SOD activity in the kidney [19] and our validation studies used an Elisa assay specific for the SOD1 isoform, supporting the notion that our findings are most likely specific to the SOD1 isomer. These findings could hold promise for the development of specific therapeutics targeting SOD1 during AKI.

The increased levels of urinary SOD1 in our patients likely represents 1 of 2 possibilities: 1) the release of SOD1 from the cytosol of damaged tubular cells or 2) an increase in SOD1 expression to combat renal injury. A few patients in our control cohort displayed high levels or urinary SOD1 despite not progressing to the primary outcome. Likewise, a small number of patients that progressed to severe AKI displayed lower levels of urinary SOD1 (Fig. 2). This fluctuation may reflect differences in systemic generation of SOD1 in response to cardiothoracic surgery in these few specific patients. When the entire patient cohort is taken collectively there is no difference in surgical parameters or patient comorbidities; however, our study was not designed or powered to analyze for changes in systemic SOD1. The active dimerized form of SOD1 is 32 kilodaltons in size, thus we cannot exclude the possibility that our measurements of urinary SOD1 concentrations could be affected by SOD1 generated in other tissue beds that enters the urine through glomerular filtration. It is known that coronary artery disease and cardiothoracic surgery both influence serum concentrations of SOD [40–42]. However, serum levels of SOD activity have not been shown to be associated with worse cardiac outcomes [42]. Furthermore, there was no statistical difference among our patient cohort regarding pre-existing diabetes mellitus, systolic heart failure, previous cardiac surgery, or the need for surgical coronary revascularization. The finding that urinary SOD1 concentrations strongly predicted the progression to severe AKI in the absence of differences in patient baseline comorbidities or the type of surgical procedure supports the hypothesis that increased urinary SOD1 concentration is driven by a renal specific mechanism. These findings warrant further investigation.

SOD1 is normally localized to the cytoplasm of renal tubule cells in the cortex, juxtaglomerular apparatus, and thick ascending limb [43, 44]. Thus, insult to the renal tubular epithelial cells could result in the release of large amounts of cytosolic SOD1 into the tubular lumen, which would then subsequently be detectable in the urine. In this scenario, urinary SOD1 concentrations would serve as a marker of renal tissue damage. Another possible explanation of our findings is that SOD1 is upregulated following acute kidney injury as a damage response mechanism. This assumption is supported by the finding that patients that progressed to more severe AKI have higher levels of urinary SOD1 concentrations and activity. The higher levels of SOD1 could reflect an exaggerated attempt to overcome oxidant damage. *Chang *et al. showed that SOD1 concentrations in rat kidney homogenates were increased at 72 h following ischemia/reperfusion surgery (IR). Furthermore, increases in SOD1 concentrations were correlated with improved renal function and attenuated cell death and apoptosis [45]. In addition, *Schneider *et al. showed that male mice with knockout of the SOD3 gene have poor renal recovery from IR surgery at 24 h, and this severe injury was attenuated in female mice likely due to upregulation of SOD1 [46]. In contrast, other animal studies have shown that renal levels of endogenous antioxidants, including SOD isoforms, are decreased at earlier timepoints following IR surgery initially, but tend to recover over time [47].

One limitation of the current study is that samples were collected at multiple time points between 0–72 h postoperatively. The various time points of collection make it difficult to extrapolate how urinary SOD1 concentration and activity are temporally related to AKI on an individual basis. It remains possible that urinary SOD1 concentrations and SOD activity vary based on the time of collection following AKI. Although our study inclusion criteria allowed collection of urinary samples at the time of diagnosis of AKI up to 72 h postoperatively, across the entire patient cohort there was no difference between the mean time of collection for controls compared to patients that progressed to severe AKI (26.4 VS 26.9 h, *p* = 0.881). These data suggest that the timing of the development of AKI postoperatively and the time of sample collection does not influence the ultimate severity or progression of AKI. An additional limitation of our study is that they were not powered to detect dynamic changes in urinary SOD1 concentrations and activity over time. Our urine samples were collected at the time of diagnosis of AKI, but we were unable to collect serial measurements of urine following the initial collection. Although we are able to correlate the urinary concentration of SOD1 early in the course of AKI with the ultimate outcome for each patient, are current study does not allow for determination of changes in urine SOD1 over time and how this relates to the trajectory of AKI severity [48]. Dynamic changes in the concentration of other urinary biomarkers have been shown to influence the biomarker’s ability to predict progression to severe AKI [9, 14]. Future studies should address the temporal relationship of urinary SOD1 concentrations and AKI to establish the ideal window of collection to predict trajectory of progression to severe AKI.

## Conclusion

We have validated urinary SOD1 concentrations and urinary SOD activity as prognostic biomarkers for AKI following cardiothoracic surgery. Our findings hold promise to identify patients likely to progress to severe renal injury following cardiothoracic injury and may help guide early intervention of therapies.

## Data Availability

All data generated or analyzed during this study are included in this article and its supplementary material files. Further enquiries can be directed to the corresponding author.

## Abbreviations

AKI: Acute kidney injury
RRT: Renal replacement therapy
SOD1: Superoxide dismutase 1
EARLYARF: Early Intervention with Erythropoietin trial
GGT: γ-Glutamyl transpeptidase
Cys-C: Cystatin C
KIM-1: Kidney injury molecule 1
NGAL: Neutrophil-gelatinase-associated lipocalin
TIMP-2: Tissue inhibitor of metalloproteinases-2
IGFBP7: Insulin-like growth factor binding protein 7
AKIN: Acute Kidney Injury Network
ESKD: End stage kidney disease
AUC: Area under the curve
